# Trends and age, sex, and race disparities in time to second primary cancer from 1990 to 2019

**DOI:** 10.1002/cam4.6785

**Published:** 2023-12-08

**Authors:** Tiffany H. Leung, Aya El Helali, Xiaofei Wang, James C. Ho, Herbert Pang

**Affiliations:** ^1^ Department of Medicine, Li Ka Shing Faculty of Medicine The University of Hong Kong Hong Kong China; ^2^ School of Public Health, Li Ka Shing Faculty of Medicine The University of Hong Kong Hong Kong China; ^3^ Department of Clinical Oncology, School of Clinical Medicine, Li Ka Shing Faculty of Medicine The University of Hong Kong Hong Kong China; ^4^ Department of Biostatistics and Bioinformatics, School of Medicine Duke University Durham North Carolina USA

**Keywords:** competing risk analysis, disparity, epidemiology, second primary cancer, SEER

## Abstract

**Background:**

Despite the growth in primary cancer (PC) survivors, the trends and disparities in this population have yet to be comprehensively examined using competing risk analysis. The objective is to examine trends in time to second primary cancer (SPC) and to characterize age, sex, and racial disparities in time‐to‐SPC.

**Methods:**

A retrospective analysis was conducted based on Surveillance, Epidemiology, and End Results (SEER). Two datasets for this study are (1) the discovery dataset with patients from SEER‐8 (1990–2019) and (2) the validation dataset with patients from SEER‐17 (2000–2019), excluding those in the discovery dataset. Patients were survivors of lung, colorectal, breast (female only), and prostate PCs.

**Results:**

The 5‐year SPC cumulative incidences of lung PC increased from 1990 to 2019, with the cumulative incidence ratio being 1.73 (95% confidence intervals [CI], 1.64–1.82; *p* < 0.001). Age disparities among all PCs remained from 2010 to 2019, and the adjusted HRs (aHRs) of all PCs were above 1.43 when those below 65 were compared with those 65 and above. Sex disparity exists among colorectal and lung PC survivors. Racial disparities existed among non‐Hispanic (NH) Black breast PC survivors (aHR: 1.11; 95% CI: 1.07–1.17; *p* < 0.001). The types of SPC vary according to PC and sex.

**Conclusions:**

Over the past three decades, there has been a noticeably shortened time‐to‐SPC among lung PC survivors. This is likely attributed to the reduced number of lung cancer deaths due to advancements in effective treatments. However, disparities in age, sex, and race still exist, indicating that further effort is needed to close the gap.

## 1 INTRODUCTION

Cancer has been one of the leading causes of death in the United States. Breast, prostate, colorectal, and lung cancers are the four most common cancers and causes of cancer‐related death.^1^ The increase in cancer survivors, aging, and growth in the population all contributed to the increasing prevalence of primary cancer (PC). In 2019, the PC population in the United States was approximately 16.9 million. It is expected to reach 22.1 million by 2030.^2^ In addition, PC survivors have a higher risk of a second primary cancer (SPC) than the general population.^3^, ^4^ Therefore, a better understanding of SPCs can potentially help improve the long‐term management of cancer survivors.

In recent years, several health disparities have been identified as potentially significant and related to the rising incidence of SPC. Data supporting the cancer disparity trends in the incidence of SPC for age, gender, and race are limited and have yet to be comprehensively investigated. Age may play a crucial role in the SPC trends. A European study identified that the SPC incidence rate was higher among males. In particular, males over 70 had an SPC incidence rate 3.25 times that of females. While for those younger than 50, the SPC incidence rate for females was twice that of males.^5^ In 2020, a global study found that nearly half of the newly diagnosed cancer cases were in the elderly, aged 65 or above.^6^ A population‐based study concluded that non‐Hispanic (NH) Black and Hispanic Breast PC survivors have a higher risk of SPC.^7^ Additionally, another study demonstrated that NH Asian or Pacific Islander prostate PC survivors had a higher standardized incidence ratio of SPC.^8^ Despite these recent findings, a comprehensive investigation of disparities in SPC based on competing risk analysis is currently lacking.

Understanding the SPC trends and the disparities can help policymakers prioritize their goals and better allocate resources to cancer survivors. To characterize SPC trends and disparities, we conducted competing risk and regression analyses using the US Surveillance, Epidemiology, and End Results (SEER) registry data collected between 1990 and 2019.

## 2 METHOD

### 2.1 Data source

#### 2.1.1 SEER database

This population‐based study was conducted using data from the SEER program in the United States. The SEER database is collected, coordinated, and deidentified by the National Cancer Institute from multiple cancer registries. Institutional review board approval and a need for informed consent were not required for our study, given that the data provided in SEER had been deidentified and are available for public use. The details of our study are reported according to the Strengthening the Reporting of Observational Studies in Epidemiology Reporting Guidelines.

#### 2.1.2 Datasets for analysis

Two datasets, the discovery dataset and the validation dataset, were used in this study to help improve the reproducibility of our findings. The discovery dataset consisted of eight cancer registries from 1990 to 2019. The validation dataset consisted of nine cancer registries from 2000 to 2019. The extracted variables included patient ID, sex, age, race, and ethnicity, year at diagnosis, types of cancer, sequence number of PCs, survival months, and status.

#### 2.1.3 Study population

Patients with curated prostate, colorectal, lung, and female‐only breast PC data were included. Those without information on survival were excluded. We defined SPC as the occurrence of PCs at least 2 months after the first PC record.^9^, ^10^ Therefore, PC patients who had experienced neither an SPC event nor death by the last follow‐up date were censored on that date. Patients were categorized into three groups according to their year at diagnosis: (1) 1990–1999, (2) 2000–2009, and (3) 2010–2019, to better understand the SPC trends. The patients in the validation dataset were separated into the latter two groups.

### 2.2 Study investigation

#### 2.2.1 Time‐to‐SPC‐event in three recent decades of four major PCs

First, to understand the trends of time‐to‐SPC events in the three time periods (1990–1999, 2000–2009, and 2010–2019), the datasets of the top four cancers were stratified by the three recent decades. Then, they were examined using competing risk analysis with death as the competing event of time‐to‐SPC‐event. Our study defines time‐to‐SPC as the time between PC and SPC diagnosis.

#### 2.2.2 Age, sex, and racial disparities in time‐to‐SPC event

Second, we assessed age, sex, and racial disparities in time‐to‐SPC‐event. Patients aged 65 and above were defined as elderly, while those below 65 were defined as young. The comparisons of young versus elderly were available for all four PCs, while the comparisons of gender were available for lung and colorectal PC. To investigate whether the magnitude of disparity has changed over time, age and sex disparity analyses were stratified by the three time periods (1990–1999, 2000–2009, and 2010–2019). As for racial disparity, patients were classified into six groups according to their race or ethnicity: Hispanic patients, NH White patients, NH Black patients, NH Asian or Pacific Islander patients, NH American Indian or American Native patients, and NH patients with unknown race. To align the follow‐up period so that the results of adjusted HRs (aHRs) between periods are comparable, data was administratively censored at 9‐year post PC diagnosis.

#### 2.2.3 Types of SPCs in four major PCs

Lastly, the types of SPCs in the four PCs were examined. While the type of cancer was only available in the ICD‐O code and primary site rather than in organs, all 328 primary sites were classified into 15 categories. The classification was mainly based on the organ systems. At the same time, common cancer sites, such as breast, colorectum, lung, and prostate, were individually separated from their organ systems and listed in the 15 categories. Considering the possibility that sex can influence the types of SPC, subgroup analysis by sex for lung PC and colorectal PC was also conducted. The details of respective SPC categories are provided in Methods S1.

### 2.3 Statistical analysis

The estimated cumulative incidence of SPC events was calculated using the Aalen–Johansen estimator^11^ for the transition probability of a multistate model. Curves of time‐to‐SPC events and death were created using the complement of the Aalen–Johansen estimate. The estimated cumulative incidence of SPC at 5 years post diagnosis was provided. To better understand the changes in cumulative incidence over time, cumulative incidence ratios, their corresponding *p*‐values and 95% confidence intervals (95% CI) were reported with the earliest time period (1990–1999) as the reference. All reported *p* values are two‐sided without adjusting for multiplicity.

Age, sex, and racial disparities were examined using multivariable Cox proportional hazards regression models. In our models, SPC is defined as our event of interest and death before SPC is considered as the competing event. The adjusted variables, which included age, sex, race/ethnicity, and income, were applied in the respective regression model when appropriate (for more details, please refer to Data S1). In the regression model for race disparity, NH White patients were defined as the reference group. The aHR, their corresponding *p*‐values and 95% CI were presented and visualized using forest plots. In the examination of age disparity, an aHR less than 1 indicates a higher risk for non‐elderly survivors while it refers to a higher risk for female and NH White patients in the examinations of sex and racial disparities, respectively. Furthermore, the types of SPC were presented by PC using a percentage bar plot.

All data analyses were performed in R 4.2.2^12^ after the raw data had been extracted from SEER*STAT 8.4.0 and managed using SAS 9.4.

## 3 RESULTS

**FIGURE 1:**
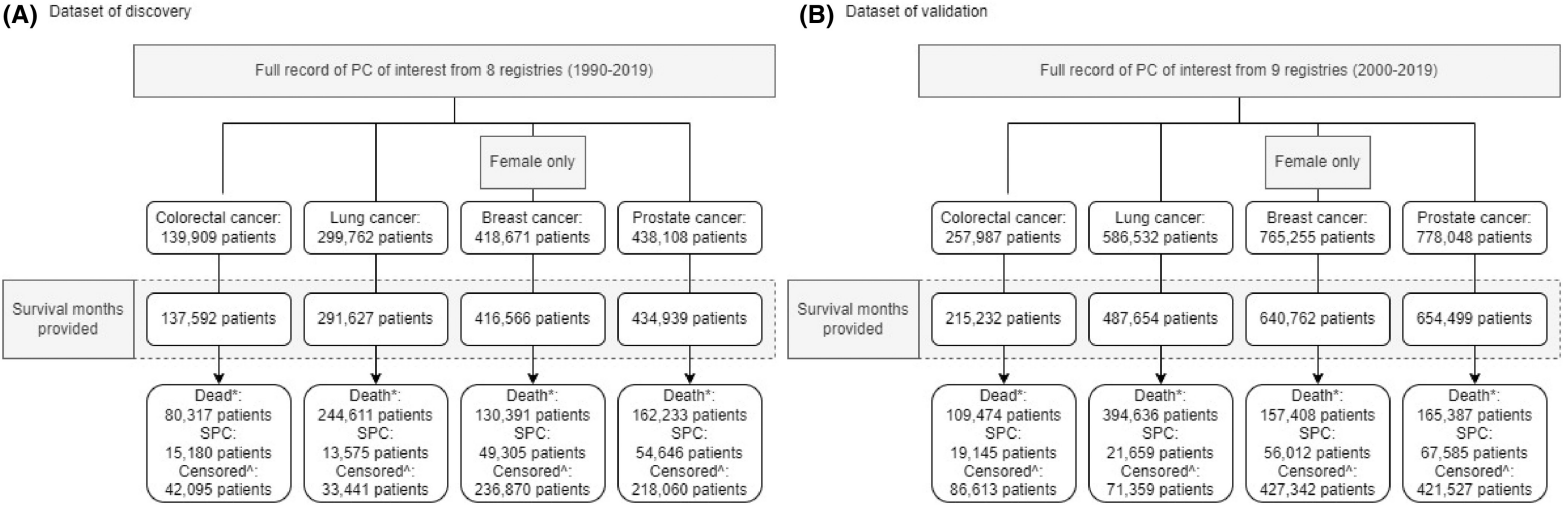
Patient inclusion process for the (A) discovery and (B) validation datasets. # Nine registries are included in the “SEER Research Data: 17 registries” database but not in the “SEER Research Data: 8 registries” database. * Death record before SPC events. ^ Patients with neither death record nor SPC event. SEER, Surveillance, Epidemiology, and End Results; SPC, second primary cancer.

Figure 1 illustrates the patient enrollment process for the discovery (Figure 1A) and validation (Figure 1B) datasets. 137,592 colorectal, 291,627 lung, 416,566 female‐only breast, and 434,939 prostate PC were included in the discovery dataset. 215,232 colorectal, 487,654 lung, 640,762 female‐only breast, and 654,499 prostate PC patients diagnosed between 2000 and 2019 were included in the validation cohort. The baseline characteristics of patients included in the discovery and validation datasets can be found in the supplementary materials (Tables S1 and S2). In addition, the results of the validation dataset can be found in Results S1 (together with Table S4, Figures S2, S3, S5, S6, S8, S9, and S11).

### 3.1 Time‐to‐SPC events

**FIGURE 2:**
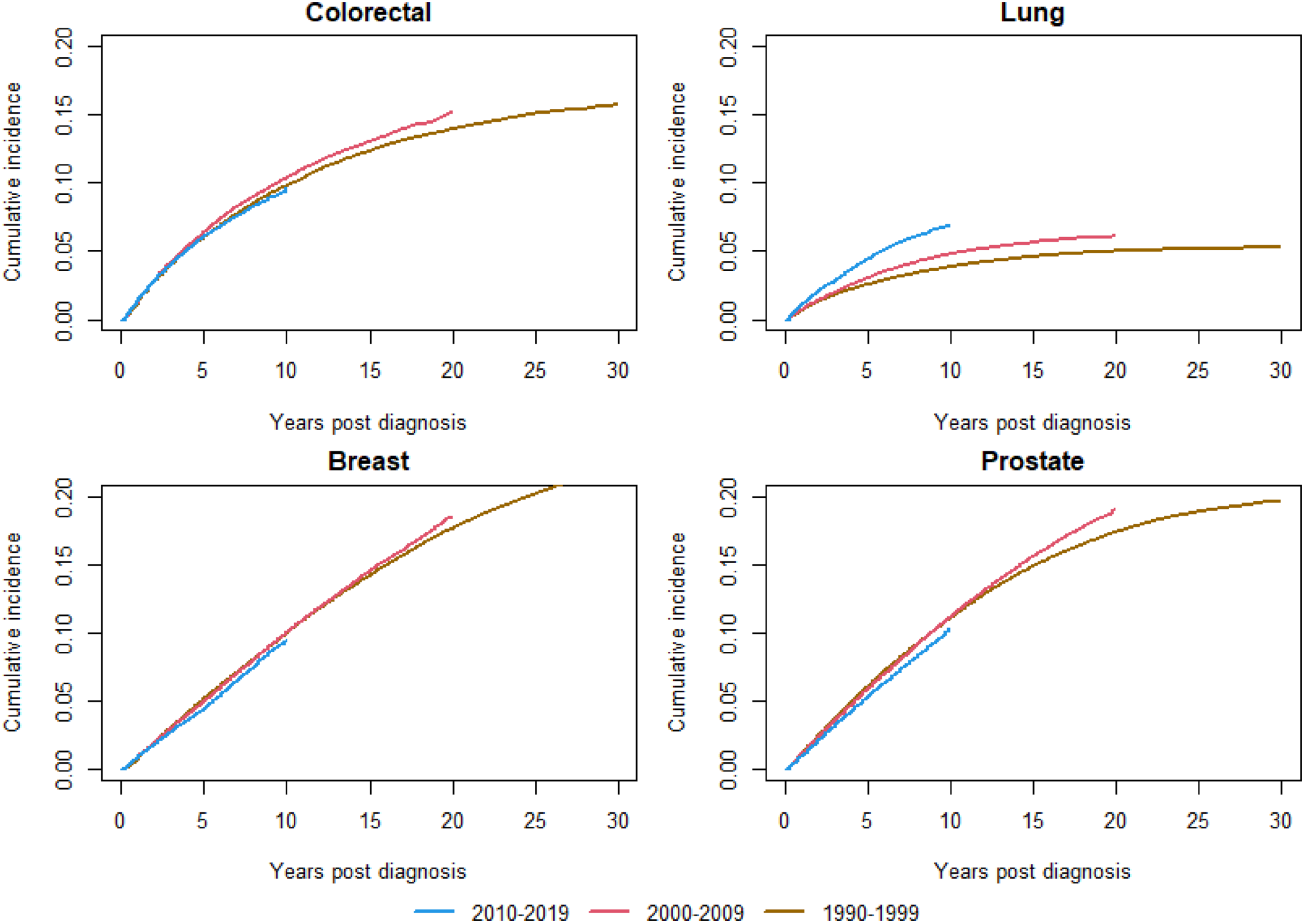
Estimated cumulative incidence curves of SPC events. SPC, second primary cancer.

Figure 2 illustrates the estimated cumulative incidence of SPC based on different PCs. The same plots zooming out to include the cumulative incidence of death as well are provided in Figure S1. In each plot, the estimated cumulative incidence of death and SPC were stratified into three groups based on the year of PC diagnosis. Among lung PC, patients diagnosed between 2010 and 2019 tended to have a shorter time‐to‐SPC than those in 2000–2009 and 1990–1999. The cumulative incidence ratio of SPC at 5 years post diagnosis of lung, breast, and prostate PC were 1.73 (95% CI: 1.64–1.82; *p* < 0.001), 0.89 (95%CI, 0.86–0.92; *p* < 0.001), and 0.90 (95%CI, 0.87–0.93; *p* < 0.001), respectively. Other cumulative incidence ratios are summarized in Table S3. We conducted additional analyses by calculating adjusted hazard ratios (aHRs) for each diagnosis period (Figure S4).

### 3.2 Age and sex disparities in time‐to‐SPC events and their trends

**FIGURE 3:**
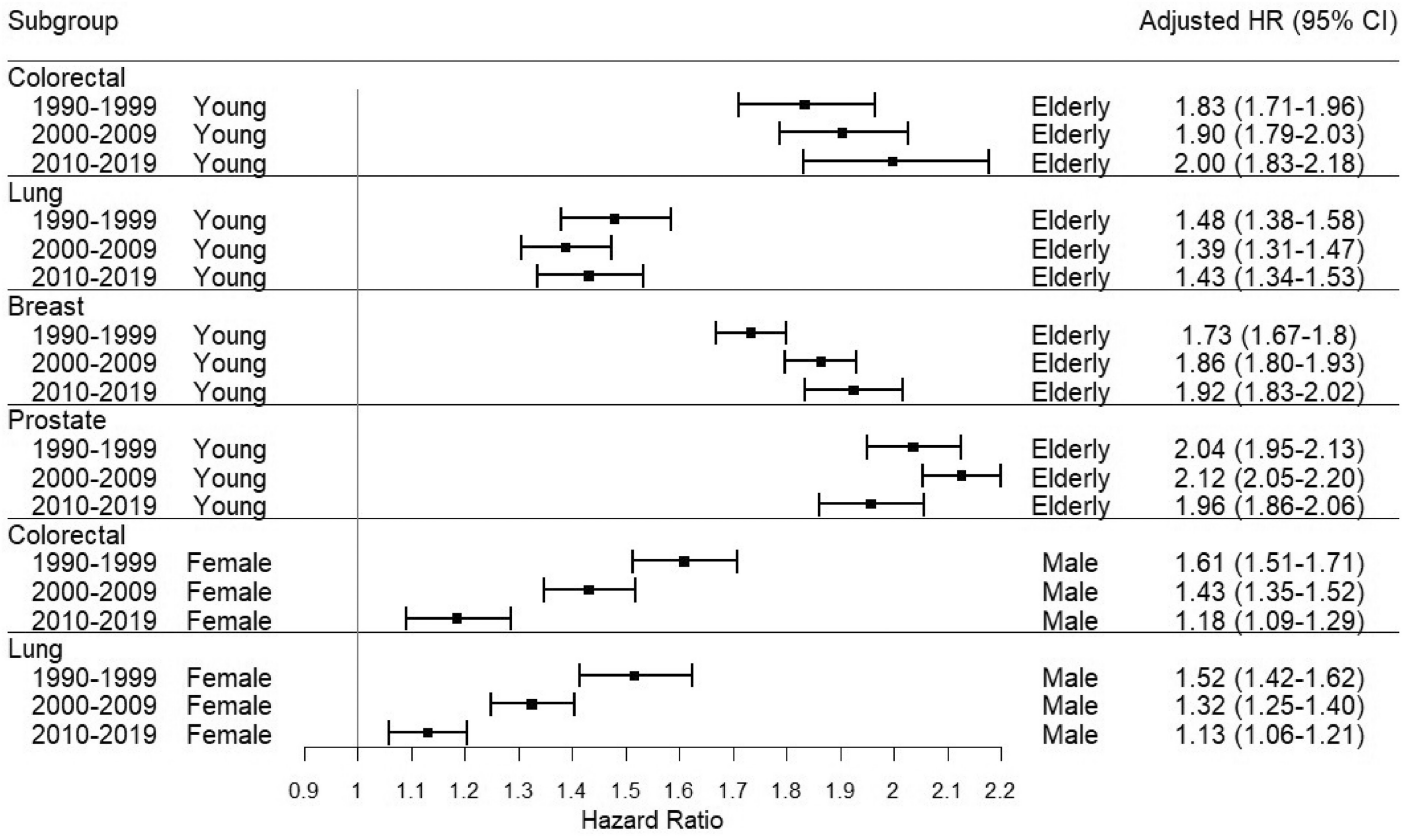
Age and sex disparities and their trends.

The aHRs for age from 1990–1999 to 2010–2019 did not indicate any clear trends, but age disparities still existed in the latest period among all four PCs. In 2010–2019, the aHR of lung PC patients was the lowest (aHR: 1.43; 95% CI: 1.34–1.53; *p* < 0.001) and the aHR of colorectal PC was the highest (aHR: 2.00; 95% CI: 1.14–1.26; *p* < 0.001). As for breast PC and prostate PC, the aHRs of 2010–2019 were 1.92 (95% CI: 1.83–2.02; *p* < 0.001) and 1.96 (95% CI: 1.86–2.06; *p* < 0.001), respectively (Figure 3). Additionally, the difference in SPC trend between males and females decreased monotonically. Despite the decreasing trend, in 2010–2019, male patients with colorectal PC (aHR: 1.18; 95% CI: 1.09–1.29; *p* < 0.001) still had a higher risk of SPC compared with their female counterparts. Similarly, male patients with lung PC (aHR: 1.13; 95% CI: 1.06–1.21; *p* < 0.001) were associated with a higher risk of SPC than females in 2010–2019 (Figure 3).

### 3.3 Racial disparities in time‐to‐SPC events

**FIGURE 4:**
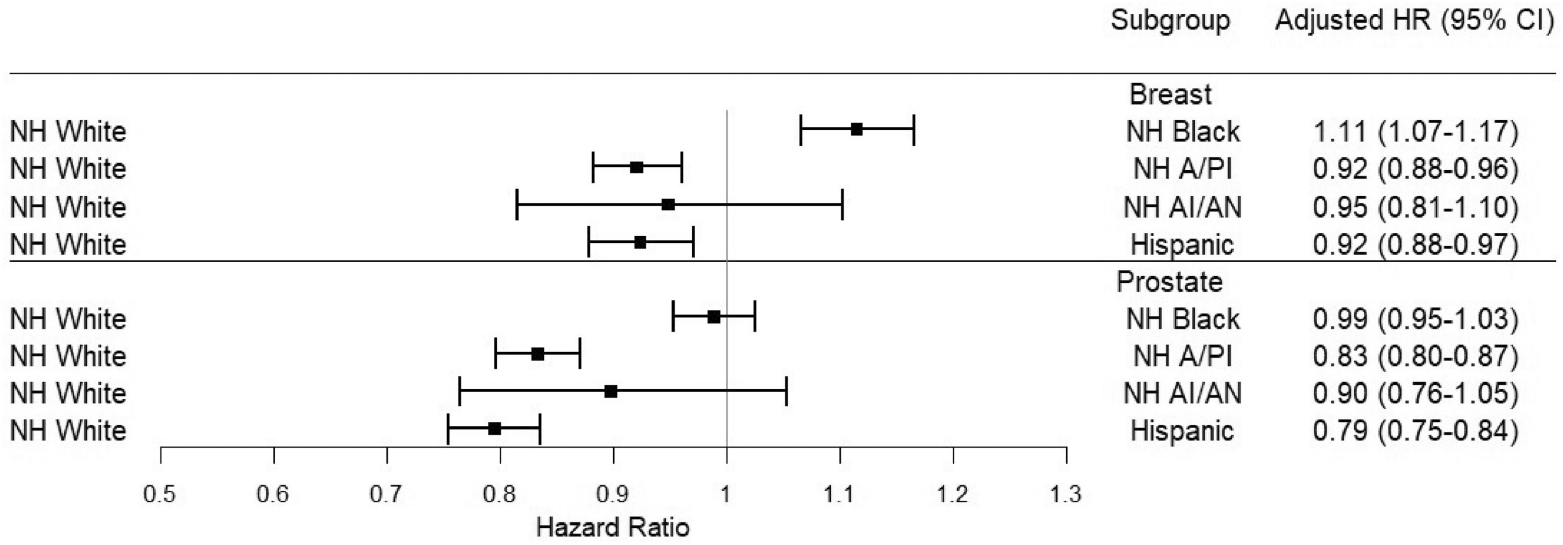
Racial disparities of breast PC and prostate PC. PC, primary cancer.

The discovery dataset demonstrated that among breast PC, NH Black patients (aHR, 1.11; 95% CI: 1.07–1.17; *p* < 0.001) had a higher risk of SPC relative to NH white patients (reference level) while NH Asian or Pacific Islander patients (aHR, 0.92; 95% CI: 0.88–0.96; *p* < 0.001) and Hispanic patients (aHR, 0.92; 95% CI: 0.88–0.97; *p* < 0.001) were associated with a lower risk comparing with NH White patients. In 2010–2019, the aHR of NH Black breast PC survivors was 1.11 (95% CI: 1.06–1.16; *p* < 0.001) overall. For prostate PC, NH Asian or Pacific Islander patients (aHR, 0.83; 95% CI: 0.80–0.87; *p* < 0.001) and Hispanic patients (aHR, 0.79; 95% CI: 0.75–0.84; *p* < 0.001) were associated with a lower risk (Figure 4). The results of colorectal and lung PC were provided in Figure S7.

### 3.4 Classification of SPCs

**FIGURE 5:**
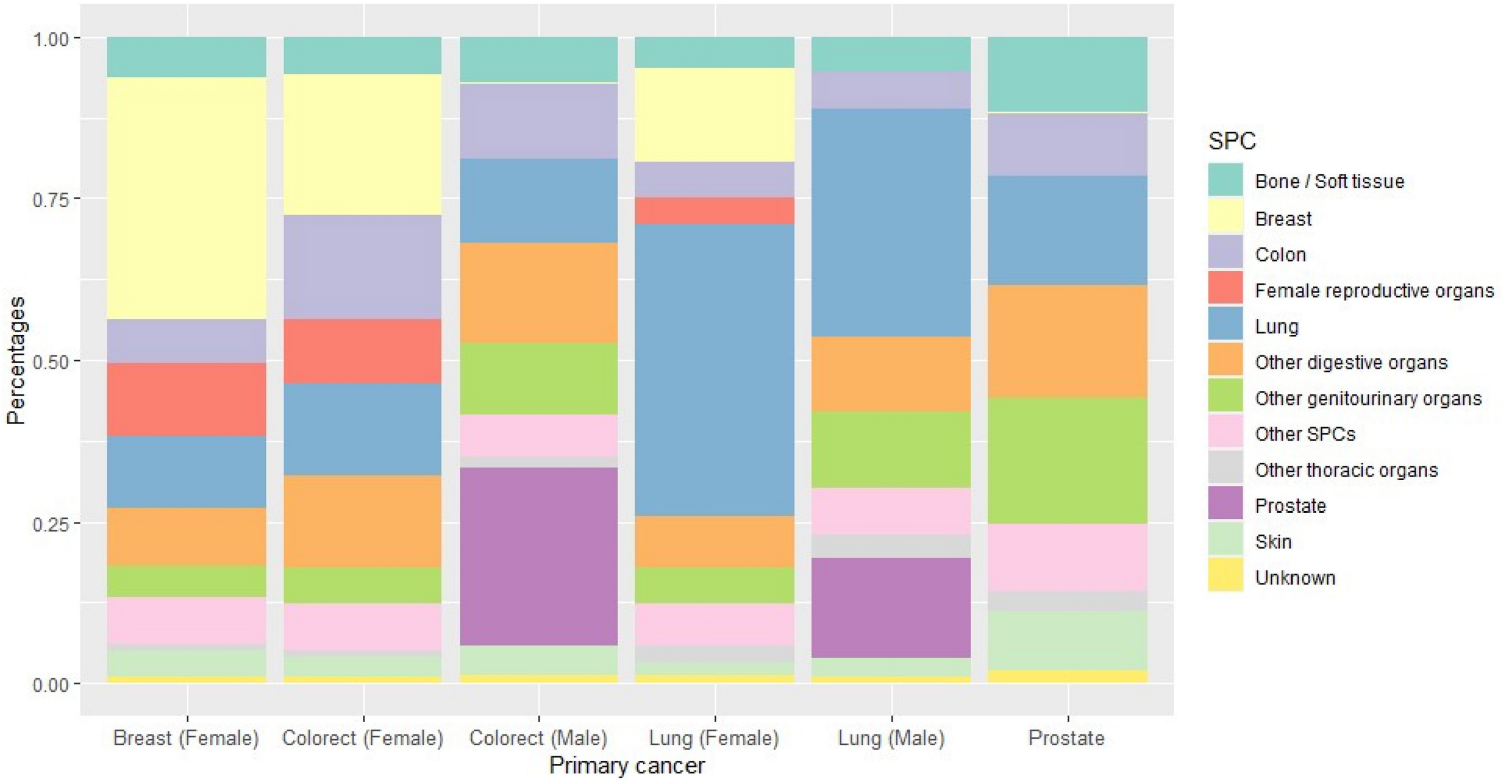
Percentages of different SPCs. * Other SPCs refer to SPCs with a percentage (1) smaller than 3% among more than three PCs and (2) no greater than 4% in any PC. This category includes endocrine, nerve system, lymph nodes, and oral. SPC, second primary cancer.

Lung cancer was the most common SPC regardless of gender (male: 35.12%; female: 45.05%) among lung PC survivors. However, this was not observed in the colorectal PC cohort, with prostate (27.61%) and breast (21.81%) being the dominant SPCs (Figure 5). We assessed the SPC type in both breast and prostate PC (Figure 5). Breast cancer (37.48%) was the most common SPC among breast PC survivors. However, most prostate PC survivors developed non‐prostate genitourinary cancers (20.09%), with bladder cancer accounting for 29.67% (Figure 5). Furthermore, we conducted an analysis of the distribution of SPCs by excluding those that originated from the same primary site as their respective PC (Figure S10). The incidence of colorectal and prostate cancer remained stable, with breast and prostate secondary cancers being the predominant malignancies in women and men with colorectal cancer, respectively. Notably, among prostate cancer cases, the majority of secondary cancers occurred in the genitourinary organs other than the prostate itself. Excluding lung SPC, breast and prostate SPCs dominate among female and male lung PC survivors, respectively. Female reproductive organ SPC and lung SPC prevail among breast PC survivors.

## 4 DISCUSSION

In this study, investigations of sex, age, and race/ethnicity disparities in time‐to‐SPC events were conducted using a nationwide database and competing risk models. Our findings suggest a shortened time‐to‐SPC event among lung PC over the past three decades. Furthermore, despite the decreasing trend of sex disparities, it remained a significant factor among the examined PC survivors. Age disparities remained among PC survivors. In terms of the types of SPC, differences were observed across various PC types and sexes.

We identified an increase in the estimated cumulative incidence of SPC in the discovery and validation datasets among lung PC survivors. One possible explanation is the reduction in cancer deaths through advances in cancer treatment.^13^, ^14^ In recent years, the positive impact of immunotherapy on lung cancer patients has been evident.^15^, ^16^ More patients survived their PCs and developed SPCs earlier in 2010–2019. More patients survived their PCs and developed SPCs earlier in 2010–2019. Therefore, the development of effective screening methods for cancer survivors is vital. In addition, slight increases in time‐to‐SPC events among breast and prostate PC survivors were observed.

Improvements in sex disparity were observed in both lung and colorectal PCs over three 3 time periods (1990–1999, 2000–2009, and 2010–2019). However, age disparity still exists in the discovery and validation datasets among colorectal and lung cancers, with male colorectal and lung cancer survivors having 1.18 and 1.13 times the likelihood of developing SPC, respectively, than females. The sex disparity in the time‐to‐SPC event cancer survivors might be attributed to the biological and behavioral differences between the two genders.^17^ A study indicated that female lung PC survivors tended to develop lung adenocarcinoma, a type of lung cancer less associated with behavioral risks but more affected by gene mutations.^18^ Since the detailed mechanism of how sex affects SPC development remains unclear, more investigation is needed.

From 2010 to 2019, age disparities existed among all four PCs in the discovery and validation datasets. Depending on the type of PC, elderly cancer survivors have a 1.43–2 times the probability of developing SPC than young cancer survivors do. The findings that the risk among the elderly is higher are consistent with previous research findings.^19^, ^20^, ^21^ Hence, the implementation of robust strategies such as bolstering the participation of elderly patients in oncology clinical trials along with augmenting their access to quality healthcare services stands as a crucial measure in mitigating the age disparity gap.^22^ In addition, conducting elderly specific studies can help develop appropriate treatments and reduce the national disease burden due to SPCs. This is crucial, as the elderly population accounts for 17.1% of the total population in 2021 and is expected to increase continuously.^23^

Due to the small sample size of some ethnicities, only overall racial disparities combined in the three time periods (1990–1999, 2000–2009, and 2010–2019) were examined. The racial disparities among NH Black female breast cancer survivors were the most evident, with a higher risk of SPC among minority groups than white in both the discovery and validation datasets. Racial disparities among NH Black patients have been observed, reported, and discussed regarding cancer care, mortality, and screening programs, but these studies mainly focused on PCs.^24^, ^25^, ^26^ While some studies pointed out that differences in genes and health behavioral risk factors, such as drinking, smoking, and physical activities, may explain this observation,^27^, ^28^ an in‐depth investigation into the reasons for the racial disparity among SPC is required.^29^

The persistent racial disparity in recent decades highlights the critical need to better understand the obstacles to improving cancer survivor care for NH Black patients. While ethnic disparity is essential in time‐to‐SPC events, social issues, such as geography, cultural differences and language barriers, are believed to be potential contributors.^30^ Therefore, different strategies, such as including more minority patients in research or insurance coverage, to address these issues among NH Black patients may also help minimize the existing racial disparity gap.^31^, ^32^

To the best of our knowledge, this study provides the first comprehensive trend analysis of time‐to‐SPC events over 30 years. Examination by subgroup can provide a more in‐depth understanding of the impact of age, sex, and race/ethnicity disparities on time‐to‐SPC events among the four PCs. The evidence provided in this manuscript highlights the clear impact of disparities, which should support policymakers and researchers in addressing strategies to target disparities and their impact on PCs. The implications of our research findings are twofold: clinical and public health. The evidence strongly suggests that a more targeted allocation of resources towards at‐risk populations, namely the elderly and NH Black population, is critical. Further, we recommend more frequent follow‐ups and screenings for these groups to facilitate early identification of SPC patients and provide timely, effective treatments. This is especially important given the observed shortened time‐to‐SPC among lung PC survivors. Our research presents compelling evidence that these measures are essential for improving patient outcomes and reducing the burden of disease in these high‐risk populations.

Another strength of this study is that it was based on the SEER database, one of the world's largest cancer databases, covering approximately 30% of the US population. As mentioned earlier, a study based on the Italian cancer registry found a higher risk of SPC among males.^5^ A German cancer registry study evaluating the risk of SPC among colorectal PC had similar findings.^33^ A Korean study found that individuals aged 40–60 are at higher risk for SPC than those under 40.^34^ Thus, generalizability of the results based on this database was ensured. The discovery and validation datasets further enhance the robustness of our findings.

This study has several limitations. First, the regression model did not include disease‐related variables such as stage and treatment. The second limitation of using the SEER database is the relative underrepresentation of minority ethnic populations compared with the national population. However, more than 309,685 and 619,640 minority groups were included in the discovery and validation datasets, respectively. This accounted for approximately 24.18% and 31.01% of the total investigated population in our discovery and validation datasets, respectively. Another limitation of the SEER database related to race and ethnicity underlies its categorization within the Asian or Pacific Islander which is questioned to be too broad.^35^, ^36^ However, considering the small sample size of the smaller subgroups, we believe that analyzing the racial disparity using “NH Asian or Pacific Islander” is appropriate. While there are now more detailed Asian groups available in recent databases, this can be a topic of investigation in the future when data becomes more mature. Additionally, it is worth noting that a pervasive limitation of the SEER database is the potential loss‐to‐follow‐up associated with patient migration outside the targeted registries.^37^ Although the US Census Bureau provides State‐to‐State migration data to estimate the annual number of migrated patients, assessing the precise magnitude of the impact on follow‐up at the patient level remains an arduous task. Last but not least, as the SEER database only captures the records of patients that were reported in the healthcare system, barriers to healthcare access or diagnostics due to racial differences or age can bias our findings.

## 5 CONCLUSIONS

In conclusion, differences in age, sex, and race over the last decade have continued to exist, although gender disparities have decreased over the past three decades. Lung cancer patients exhibited a shortened time‐to‐SPC. More effective lung cancer‐related therapies, such as the introduction of immunotherapy, may increase the chance of PC survival and therefore the possibility of developing SPC. Despite advances in cancer treatment, more research on cancer survivors is needed to further reduce the disparity and better cope with their growing numbers.

## AUTHOR CONTRIBUTIONS

**Tiffany H. Leung:** Conceptualization (equal); data curation (lead); formal analysis (lead); investigation (equal); methodology (equal); writing – original draft (equal). **Aya El Helali:** Investigation (equal); writing – review and editing (equal). **Xiaofei Wang:** Investigation (equal); methodology (equal); writing – review and editing (equal). **James C. Ho:** Investigation (equal); supervision (equal); writing – review and editing (equal). **Herbert Pang:** Conceptualization (equal); formal analysis (supporting); investigation (equal); supervision (equal); writing – original draft (equal).

## FUNDING INFORMATION

This study was partially supported by the University Postgraduate Fellowships of HKU Foundation (THL).

## CONFLICT OF INTEREST STATEMENT

Dr. Pang reports personal fees from Genentech, and Roche stocks, outside the submitted work.

## Supporting information


Data S1:

## Data Availability

The data that support the findings of this study are openly available at https://seer.cancer.gov/ The SEER database agreement was signed for access to the SEER database.
