# Supplementary material for: Trends and age, sex, and race disparities in time to second primary cancer from 1990 to 2019
Source: Cancer Med. 2023 Dec 8;12(24):22316–24. doi: 10.1002/cam4.6785 (PMC10757087; doi:10.1002/cam4.6785)
Supplement: Supplementary file 1 — Data S1: [file CAM4-12-22316-s001.docx]

Content

Methods S1. Category of SPCs p.02

Methods S2. Definitions of adjusted variables p.02

Methods S3. Regression models p.02

Methods S4. Competing risk models p.02

Results S1. Results of validation dataset p.03

Table S1. Characteristics of patients in discovery dataset p.04

Table S2. Characteristics of patients in validation dataset p.04

Table S3. Estimated incidence of SPC at 5 years post diagnosis (Discovery dataset) p.05

Table S4. Estimated incidence of SPC at 5 years post diagnosis (Validation dataset) p.05

Figure S1. Cumulative incidence curves of SPC events and death (Discovery dataset) p.06

Figure S2. Cumulative incidence curves of SPC events (Validation dataset) p.07

Figure S3. Cumulative incidence curves of SPC events and death (Validation dataset) p.08

Figure S4. Disparities in year at diagnosis (Discovery dataset) p.09

Figure S5. Disparities in year at diagnosis (Validation dataset) p.09

Figure S6. Age and sex disparities and their trends (Validation dataset) p.10

Figure S7. Racial disparities of colorectal PC and lung PC (Discovery dataset) p.10

Figure S8. Racial disparities (Validation dataset) p.11

Figure S9. Percentages of different SPCs (Validation dataset) p.11

Figure S10. Percentages of different SPCs after excluding same primary sites (Discovery dataset) p.12

Figure S11. Percentages of different SPCs after excluding same primary sites (Validation dataset) p.12

Methods S1. Category of SPCs

| **Category** | **ICD code** |
| --- | --- |
| Bone / Soft tissue | C40 - C42, C49 |
| Breast | C50 |
| Colon | C18 |
| Female reproductive organs | C51 - C58 |
| Lung | C34 |
| Other digestive organs | C15 - C17, C20 - C26, C42 |
| Other genitourinary organs | C60, C62 - C69 |
| Other SPCs | Endocrine (C73 - C75); Lymph node (C77);  Nerve system (C47, C48, C69 - C72); Oral (C00 - C14) |
| Other thoracic organs | C30 - C33, C37 - C39 |
| Prostate | C61 |
| Skin | C44, C76 |
| Unknown | C80 |

Methods S2. Definitions of adjusted variables

Race: (1) Non-Hispanic White and (2) Others, which include non-Hispanic Black, non-Hispanic Asian or Pacific Islander, non-Hispanic American Indian or American Native and non-Hispanic unknown race.

Annual income: (1) less than USD 35,000, (2) USD 35,000 - USD 55,000, (3) USD 55,000 – USD 75,000, (4) above USD 75,000 and (5) Unknown

Methods S3. Multivariable regression models

| Disparity to be examined | PCs | Adjusted variables |
| --- | --- | --- |
| Age | Colorectal PC and Lung PC | Race and sex |
| Age | Breast PC and prostate PC | Race |
| Sex | Colorectal PC and Lung PC | Race and age |
| Race | Colorectal PC and Lung PC | Year at diagnosis, age, sex and income |
| Race | Breast PC and prostate PC | Year at diagnosis, age and income |

Methods S4. Competing risk models

In this study, two types of competing risk models were used: (1) Aalen-Johansen estimator for the transition probability of a multi-state model was used to plot the time-to-SPC-curves. Time to event, event status and year at diagnosis were applied to this model with the survival curves being calculated using *survfit()* function and plotted using *plot()* function. (2) Multivariable Cox proportional hazard regression models was used to calculate the adjusted hazard ratios. Applied variables other than time to event and event status for respective model were summarized in eMethods 2. More details including descriptions and comparison of these two competing risk models can be found in the R package instruction*.

* Therneau, T., Crowson, C., & Atkinson, E. (2020). Multi-state models and competing risks. *CRAN-R (https://cran. r-project. org/web/packages/survival/vignettes/compete. pdf)*.

Results S1. Results of validation dataset

Time to SPC event in 3 recent decades of 4 major PCs

In the validation dataset (Figure S2, Figure S3 and Table S4), the 5-year SPC estimated cumulative incidence ratio of lung, breast and prostate cancer were 1.40 (95% CI, 1.36-1.45), 0.92 (95% CI, 0.90-0.94) and 0.91 (95% CI, 0.89-0.93).

Age and sex disparities in time to SPC event and their trends

The validation dataset (Figure S4) illustrated that the elderly patient population had a higher risk of SPC across both decades. In addition, the aHRs of prostate PC were the highest among all PCs. Regarding the gender disparity, male colorectal and lung PC survivors were at a higher risk of SPC in both decades.

Racial disparities in time to SPC event

The validation dataset emphasized the results reported in the discovery dataset (Figure S6). The NH Black race with breast PC (aHR: 1.17; 95% CI, 1.13-1.20; P<0.001) demonstrated a higher risk of SPC relative to the NH white (reference arm)

Types of SPCs in 4 major PCs

The validation dataset showed comparable results to that observed in the discovery dataset (Figure S7).

Table S1. Characteristics of patients in discovery dataset

|  |  | **Colorectal** | **Lung** | **Breast** | **Prostate** |
| --- | --- | --- | --- | --- | --- |
|  |  | N=137592 | N=291627 | N=416566 | N=434939 |
| **Age group** | |  |  |  |  |
|  | 1-64 | 51086 (37.13) | 100537 (34.47) | 249405 (59.87) | 162177 (37.29) |
|  | 65+ | 86506 (62.87) | 191090 (65.53) | 167161 (40.13) | 272762 (62.71) |
| **Sex** | |  |  |  |  |
|  | Male | 69446 (50.47) | 158588 (54.38) | 0 (0) | - |
|  | Female | 68146 (49.53) | 133039 (45.62) | 416566 (100) | - |
| **Race** | |  |  |  |  |
|  | NH White | 101149 (73.51) | 227420 (77.98) | 311722 (74.83) | 330748 (76.04) |
|  | NH Black | 11138 (8.09) | 23336 (8) | 31744 (7.62) | 44880 (10.32) |
|  | NH Asian/Pacific islander | 14877 (10.81) | 25993 (8.91) | 40323 (9.68) | 28416 (6.53) |
|  | NH American Indian/Alaska Native | 1038 (0.75) | 1533 (0.53) | 2916 (0.7) | 2137 (0.49) |
|  | NH Unknown | 467 (0.34) | 344 (0.12) | 1376 (0.33) | 4485 (1.03) |
|  | Hispanic | 8923 (6.49) | 13001 (4.46) | 28485 (6.84) | 24273 (5.58) |
| **Years at diagnosis** | |  |  |  |  |
|  | 1990-1999 | 47129 (34.25) | 94665 (32.46) | 120546 (28.94) | 132734 (30.52) |
|  | 2000-2009 | 47688 (34.66) | 98179 (33.67) | 136618 (32.8) | 154966 (35.63) |
|  | 2010-2019 | 42775 (31.09) | 98783 (33.87) | 159402 (38.27) | 147239 (33.85) |

Table S2. Characteristics of patients in validation dataset

|  |  | **Colorectal** | **Lung** | **Breast** | **Prostate** |
| --- | --- | --- | --- | --- | --- |
|  |  | N=215232 | N=487654 | N=640762 | N=654499 |
| **Age group** | |  |  |  |  |
|  | 1-64 | 89293 (41.49) | 168915 (34.64) | 388341 (60.61) | 269466 (41.17) |
|  | 65+ | 125939 (58.51) | 318739 (65.36) | 252421 (39.39) | 385033 (58.83) |
| **Sex** | |  |  |  |  |
|  | Male | 110369 (51.28) | 259473 (53.21) | (0) | - |
|  | Female | 104863 (48.72) | 228181 (46.79) | 640762 (100) | - |
| **Race** | |  |  |  |  |
|  | NH White | 143243 (66.55) | 371010 (76.08) | 431771 (67.38) | 432483 (66.08) |
|  | NH Black | 27635 (12.84) | 55106 (11.3) | 69927 (10.91) | 101277 (15.47) |
|  | NH Asian/Pacific islander | 15661 (7.28) | 26037 (5.34) | 47814 (7.46) | 27224 (4.16) |
|  | NH American Indian/Alaska Native | 1222 (0.57) | 2432 (0.5) | 2954 (0.46) | 1976 (0.3) |
|  | NH Unknown | 1356 (0.63) | 808 (0.17) | 4047 (0.63) | 17495 (2.67) |
|  | Hispanic | 26115 (12.13) | 32261 (6.62) | 84249 (13.15) | 74044 (11.31) |
| **Years at diagnosis** | |  |  |  |  |
|  | 2000-2009 | 111285 (51.7) | 248126 (50.88) | 299512 (46.74) | 334281 (51.07) |
|  | 2010-2019 | 103947 (48.3) | 239528 (49.12) | 341250 (53.26) | 320218 (48.93) |

Table S3. Estimated incidence of SPC at 5 years post diagnosis (Discovery dataset)

| Year at Diagnosis | Colorectal cancer | Lung cancer | Breast cancer | Prostate cancer |
| --- | --- | --- | --- | --- |
| 1990-1999 | 6.10% | 2.58% | 5.22% | 6.13% |
| 2000-2009 | 6.43% | 3.09% | 5.01% | 5.94% |
| 2010-2019 | 6.10% | 4.46% | 4.46% | 5.36% |
| CIR^#^ (95% CI) | 1.00 (0.95-1.06) | 1.73 (1.64-1.82) | 0.85 (0.82-0.88) | 0.87 (0.85-0.90) |
| CIR^^^ (95% CI) | 0.95 (0.90-1.00) | 1.44 (1.37-1.52) | 0.89 (0.86-0.92) | 0.90 (0.87-0.93) |

^#^ Cumulative incidence ratio (2010-2019 versus 1990-1999)

^^^ Cumulative incidence ratio (2010-2019 versus 2000-2009)

Table S4. Estimated incidence of SPC at 5 years post diagnosis (Validation dataset)

| Year at Diagnosis | Colorectal cancer | Lung cancer | Breast cancer | Prostate cancer |
| --- | --- | --- | --- | --- |
| 2000-2009 | 6.14% | 2.99% | 4.99% | 6.17% |
| 2010-2019 | 6.24% | 4.20% | 4.60% | 5.61% |
| CIR^^^ (95% CI) | 1.02 (0.98-1.05) | 1.40 (1.36-1.45) | 0.92 (0.90-0.94) | 0.91 (0.89-0.93) |

^^^ Cumulative incidence ratio (2010-2019 versus 2000-2009)

Figure S1. Cumulative incidence curves of SPC events and death (Discovery dataset)


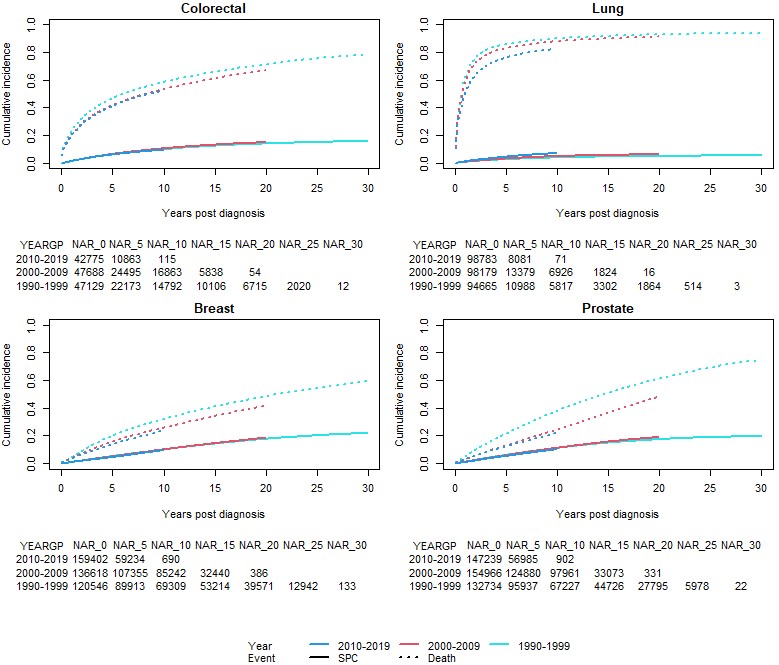


Figure S2. Cumulative incidence curves of SPC events (Validation dataset)


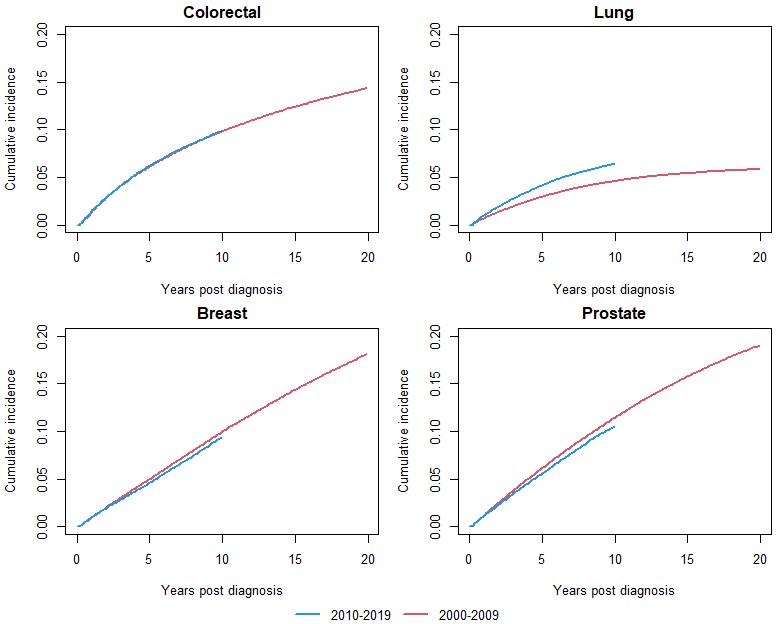


Figure S3. Cumulative incidence curves of SPC events and death (Validation dataset)


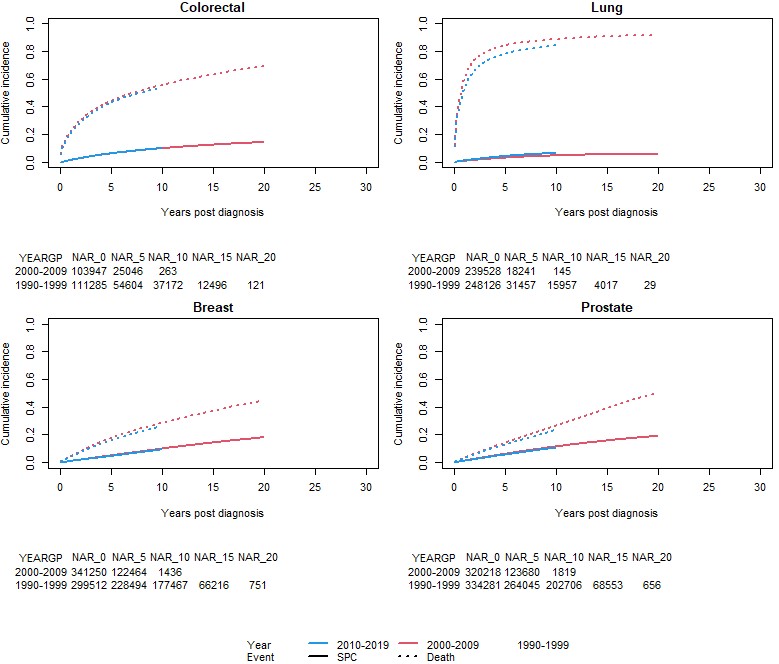


* Range of x-axis is kept to be consistent with the main figure

Figure S4. Disparities in year at diagnosis (Discovery dataset)


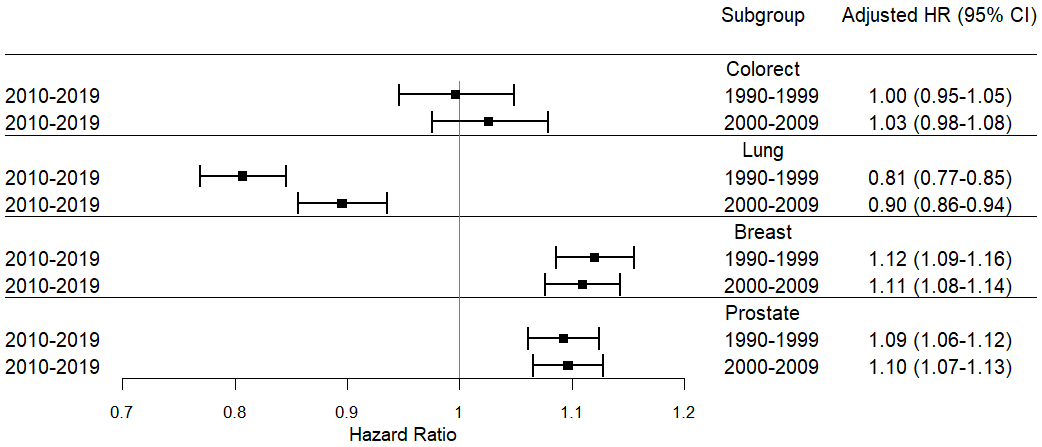


Figure S5. Disparities in year at diagnosis (Validation dataset)


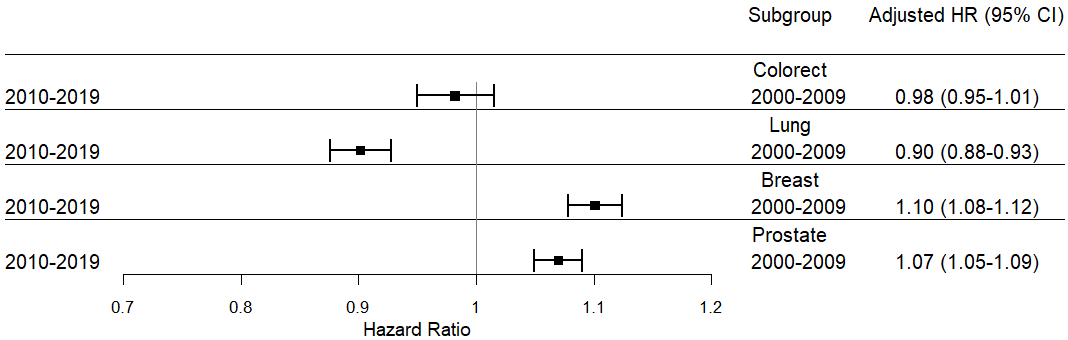


Figure S6. Age and sex disparities and their trends (Validation dataset)


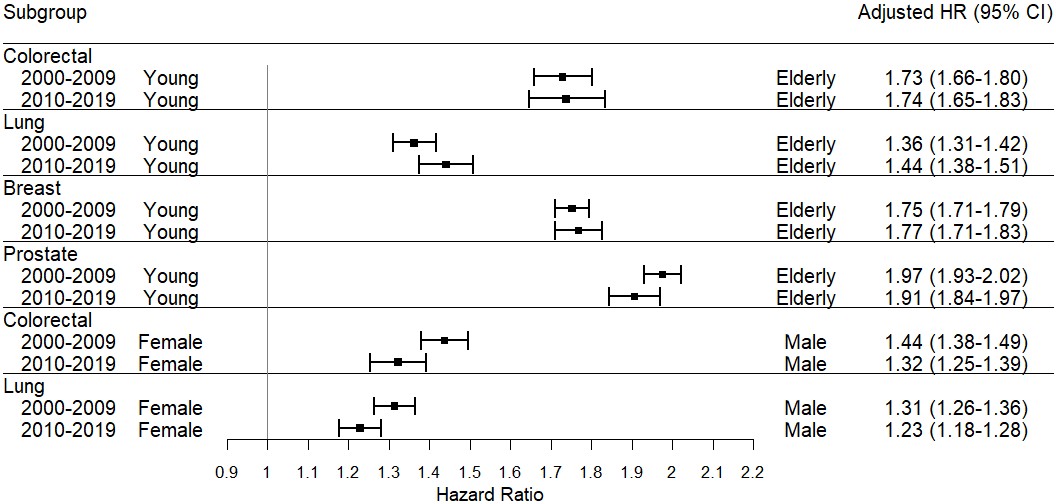


Figure S7. Racial disparities of colorectal PC and lung PC (Discovery dataset)


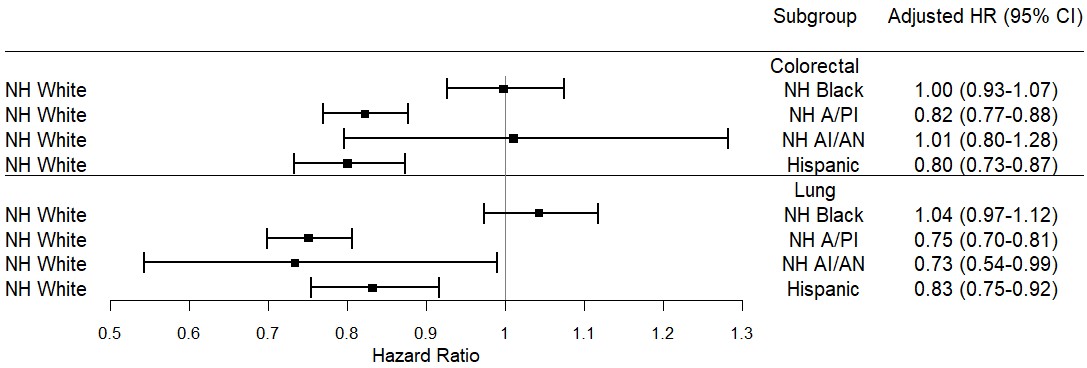


Figure S8. Racial disparities (Validation dataset)


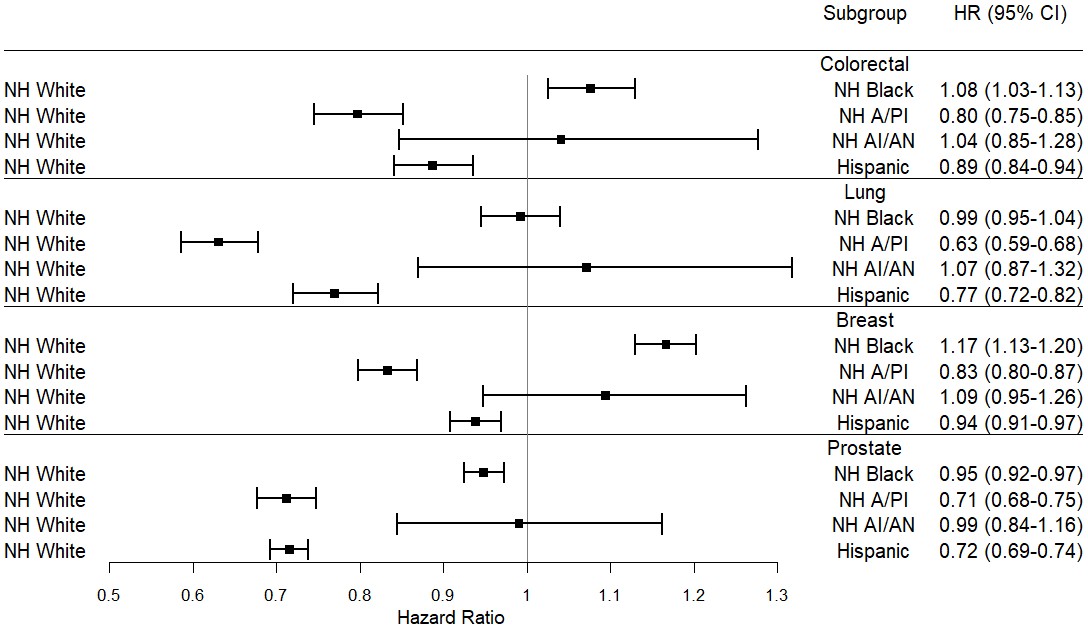


Figure S9. Percentages of different SPCs (Validation dataset)


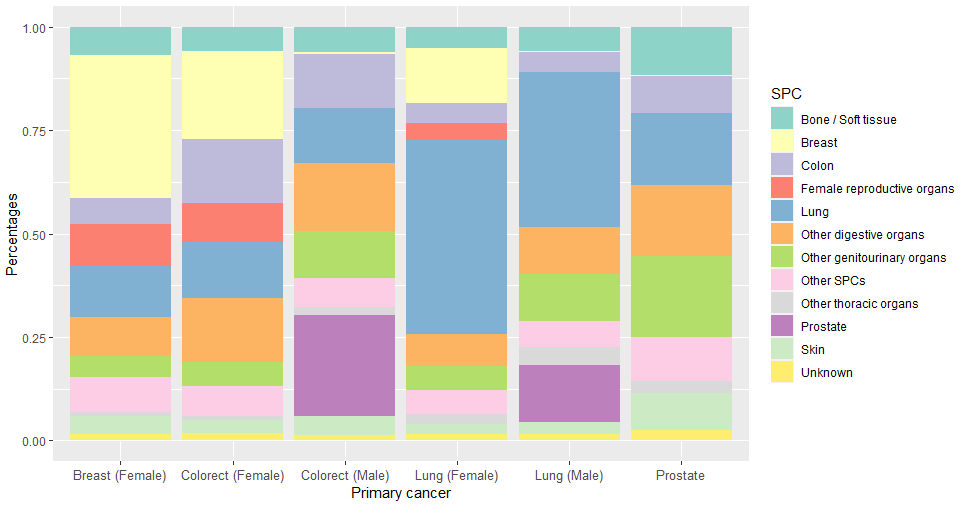


* Other SPCs refers to SPCs with (1) the number of percentage smaller than 3 % among more than 3 PCs and (2) the number of percentage is no greater than 4% in any PC. This category includes Endocrine, Nerve system, Lymph nodes and Oral.

Figure S10. Percentages of different SPCs after excluding same primary sites (Discovery dataset)


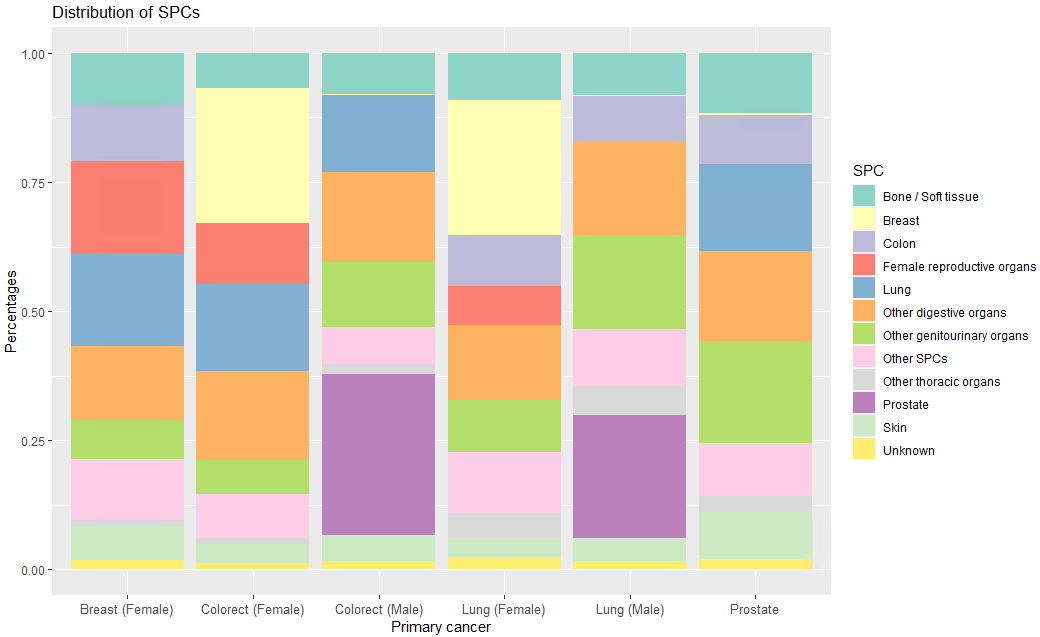


* Other SPCs refers to SPCs with (1) the number of percentage smaller than 3 % among more than 3 PCs and (2) the number of percentage is no greater than 4% in any PC. This category includes Endocrine, Nerve system, Lymph nodes and Oral.

Figure S11. Percentages of different SPCs after excluding same primary sites (Validation dataset)


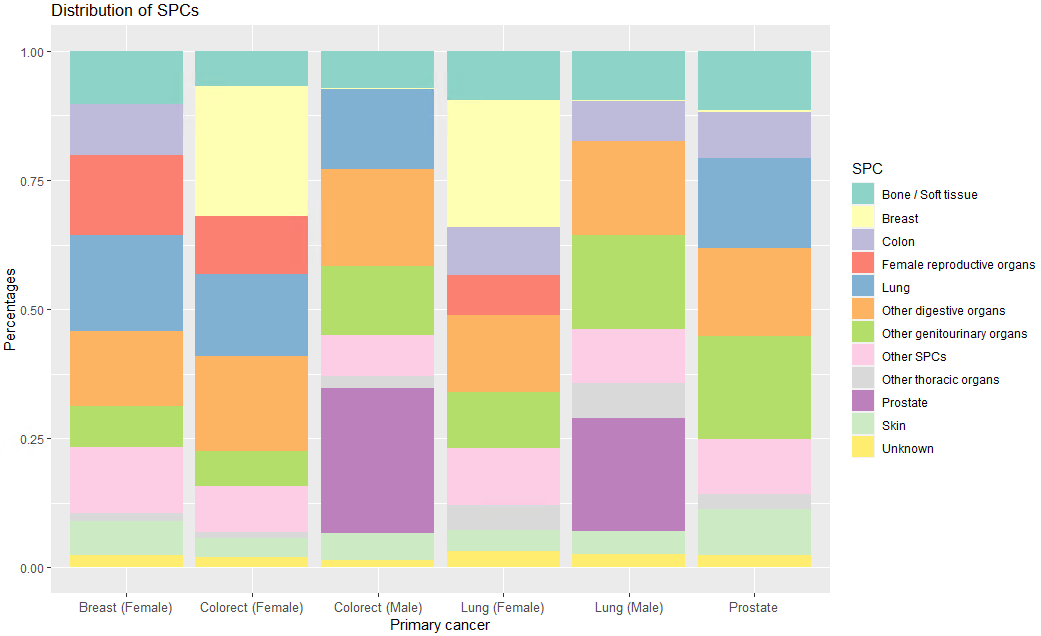


* Other SPCs refers to SPCs with (1) the number of percentage smaller than 3 % among more than 3 PCs and (2) the number of percentage is no greater than 4% in any PC. This category includes Endocrine, Nerve system, Lymph nodes and Oral.
